# Upscaling Perovskite Photovoltaics: from 156 cm^2^ Modules to 0.73 M^2^ Panels

**DOI:** 10.1002/advs.202416316

**Published:** 2025-04-26

**Authors:** Hafez Nikbakht, Paolo Mariani, Luigi Vesce, Francesco Di Giacomo, Enrico Leonardi, George Viskadouros, Emmanuel Spiliarotis, Konstantinos Rogdakis, Sara Pescetelli, Antonio Agresti, Sebastiano Bellani, Francesco Bonaccorso, Emmanuel Kymakis, Aldo Di Carlo

**Affiliations:** ^1^ CHOSE – Centre for Hybrid and Organic Solar Energy University of Rome Tor Vergata via del Politecnico1 Rome 00133 Italy; ^2^ Solertix via Eusebio Chini 15 Rome 00147 Italy; ^3^ GreatCell Solar Italia SRL viale Castro Pretorio 122 Rome 00185 Italy; ^4^ Department of Electrical & Computer Engineering Hellenic Mediterranean University Heraklion Crete GR71410 Greece; ^5^ Department of Mineral Resources Engineering Technical University of Crete Chania Crete 731 00 Greece; ^6^ Institute of Emerging Technologies University Research and Innovation Center HMU Heraklion Crete 71410 Greece; ^7^ BeDimensional S.p.A. Via Lungotorrente Secca, 30R Genova 16163 Italy; ^8^ Istituto di Struttura della Materia‐Consiglio Nazionale delle Ricerche Roma (CNR‐ISM) via del Fosso del Cavaliere 100 Rome 00133 Italy

**Keywords:** perovskite solar modules, perovskite solar panel, photovoltaic, solar cell, up scaling

## Abstract

This study tackles the challenge of upscaling perovskite solar modules (PSMs) to attain high power conversion efficiencies (PCEs) suitable for industrial applications. Through systematic experimentation, a remarkable PCE of 17.68% for PSMs fabricated on a substrate with dimensions of 15.6 cm×15.6 cm is achieved. By refining the cell interconnection design, a geometric fill factor (GFF) of 96.4% is obtained, marking a significant milestone in bridging the performance gap between individual cells and modules. Building on this success, it is fabricated and tested large‐area perovskite solar panels (PSPs) with an area of 0.73 m^2^, integrating the optimized PSMs. This work not only demonstrates the feasibility of large‐scale perovskite‐based photovoltaic systems but also sets a new benchmark for the PCE and scalability of these technologies, paving the way for their practical application in renewable energy generation.

## Graphical abstract

From lab to sunlight: perovskite photovoltaics are scaled from 156 cm² large area modules to 0.73 m² panels. With 17.68% efficiency at module level and a stron 12% PCE in outdoor conditions, this advance represents a significant step toward real‐world deployment, paving the way from innovation to practical application.

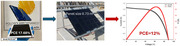

## 1 Introduction

In the quest for sustainable and efficient energy sources, photovoltaics (PVs) has been at the forefront of innovation and development. Among the various PV technologies, perovskite solar cells (PSCs) have garnered significant attention over the past decade. Their appeal lies in their remarkable facile material processability, tunable bandgap, low exciton binding energy, high absorption coefficients, and long carrier diffusion lengths. These attributes offer a compelling combination of cost‐effective manufacturing and high‐power conversion efficiency (PCE).^[^ ^1^, ^2^, ^3^ ^]^ Such advantages make PSCs particularly well‐suited for scalable, solution‐based fabrication methods.^[^ ^1^, ^3^ ^]^ The projected levelized cost of energy (LCOE) for single‐junction perovskite solar modules (PSMs) is ≈3–6 ¢/kWh,^[^ ^4^, ^5^ ^]^ which is lower than the LCOE for silicon solar modules, which ranges from 3.4 to 6.2 ¢/kWh.^[^ ^6^, ^7^, ^8^ ^]^ Additionally, the minimum sustainable price for single‐junction PSMs is ≈0.18–0.21 $/W,^[^ ^7^, ^9^ ^]^ making them more cost‐effective compared to advanced silicon solar modules, which have a manufacturing cost of ≈0.25–0.27 $/W.^[^ ^7^, ^9^ ^]^ Even at smaller production scales, perovskite solar panels (PSPs) can achieve a competitive LCOE that rivals other PV technologies.^[^ ^5^ ^]^

**Figure 1:**
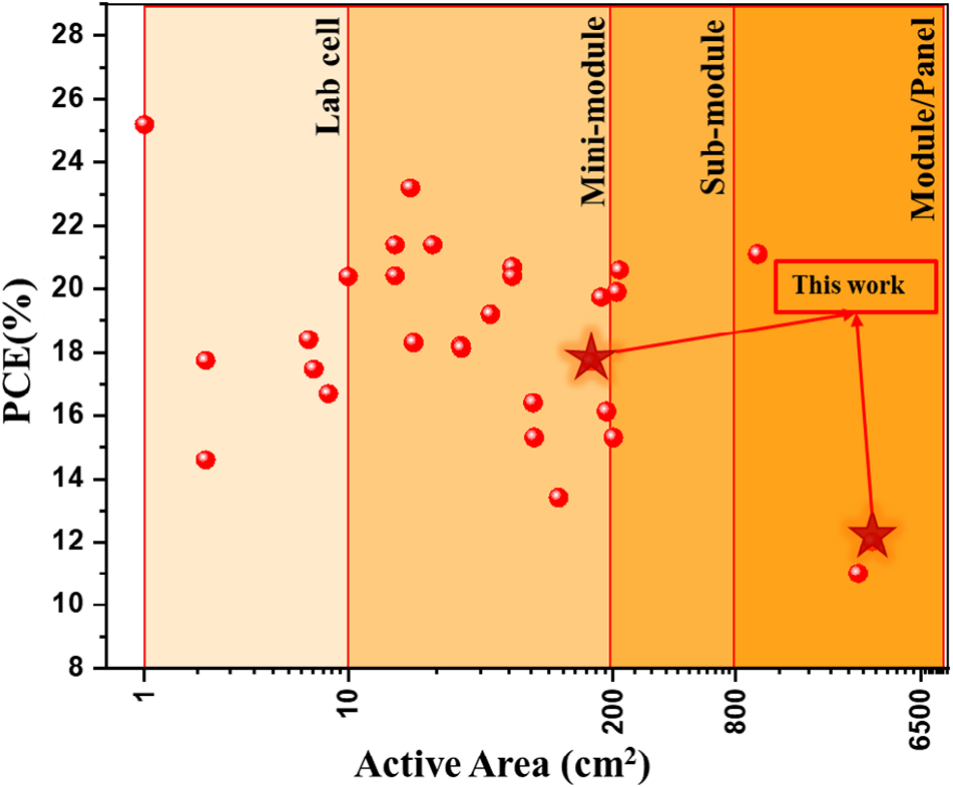
Comparison of PCEs across various sizes, *i.e*., PSMs active area, published in scientific literature.^[^ ^9^, ^12^, ^14^, ^15^, ^16^, ^17^, ^18^, ^19^ ^]^

The PV industry's key challenge is to develop a reliable and efficient process for designing and manufacturing solar modules that achieve high PCEs while ensuring extended lifetime stability of over 20 years. To date, only a few reports document the successful fabrication of large‐scale PSPs. Y. Hu et al. described the fabrication of a 1 m^2^ PSP based on 10 × 10 cm^2^ PSMs, but the panel was not functional after fabrication despite claims of process reproducibility.^[^ ^10^ ^]^ L.A. Castriotta et al. demonstrated a 0.55 m^2^ PSP based on 20 × 20 cm^2^ PSMs, but the panel did not achieve a satisfactory PCE due to issues in the encapsulant lamination process.^[^ ^11^ ^]^ Conversely, S. Pescetelli, A. Agresti, G. Viskadouros et al. reported the first outdoor‐operating solar farm consisting of nine PSPs (called GRAPE), each with an area of ≈0.5 m^2^ and consisting of 40 PSMs (11 cm × 11 cm), achieving an average PCE of 11% on panel active area.^[^ ^12^ ^]^ While significant progress has been made in advancing single‐junction PSMs, most efforts have been limited to smaller scale. Although considerable academic efforts have focused on improving the PV performance of single‐junction PSCs, the industrialization of perovskite‐based PVs requires the development of efficient, reliable, scalable and cost‐effective manufacturing methods.^[^ ^13^ ^]^ These methods must bridge the gap from laboratory successes to practical, large‐scale production, while also providing a benchmark against existing commercial PV technologies. Currently, PSCs have achieved certified PCE of 26.1% on very small active area, namely 0.057 cm^2^. However, for 1 cm^2^‐active area PSCs, the record‐certified PCE stands at 21.6%, a figure that has remained largely unchallenged since 2019.^[^ ^14^ ^]^ This PCE gap is mitigated when scaling up the device dimensions is carried out by implementing a panel configuration composed of mini‐modules connected in series or parallel (as reported by groups of the authors of this work ^[^ ^12^ ^]^). In fact, this configuration is greatly preferred over a single large module because, in real‐world conditions, interconnected perovskite modules provide superior efficiency, scalability, reliability, and thermal management, making them a more practical choice.^[^ ^12^ ^]^ (to further discussion see S.I. section: Perovskite solar panel architecture) **Figure** **1** depicts the PCEs of PSMs across their active area, indicating a notable PCE gap for large‐aperture area devices when compared to silicon solar cells (PCE >20%).^[^ ^9^, ^12^, ^14^, ^15^, ^16^, ^17^, ^18^, ^19^ ^]^ Thus, this analysis underscores the ongoing challenges in scaling perovskite‐based PV technology to compete with established PV technologies.

In general, the PCE of PSMs tends to decrease as the active area increases, a trend influenced by several key factors. First, imperfections in film coverage and homogeneity result in a wide distribution of PCEs. Additionally, the increased defect density within the perovskite layers hinders the extraction and transport of photoinduced carriers, further compromising device performance. This issue is further exacerbated by the rise in series resistance (Rs), primarily due to the high sheet resistance of transparent conductive electrodes (TCE) in large‐area PSMs. To mitigate resistance losses, large cells are divided into smaller sub cells connected in series.^[^ ^24^, ^25^ ^]^ Optimizing the design of large‐area perovskite‐based PVs is essential not only to achieve high PCE on active area but also to maximize the geometrical fill factor (GFF). In particular, this approach aims to achieve PCE over aperture area that can compete with commercially available technologies, e.g. crystalline Si solar cells (certified record PCE = 26.81% for n‐type heterojunction configuration from LONGI).^[^ ^14^ ^]^)

**Figure 2:**
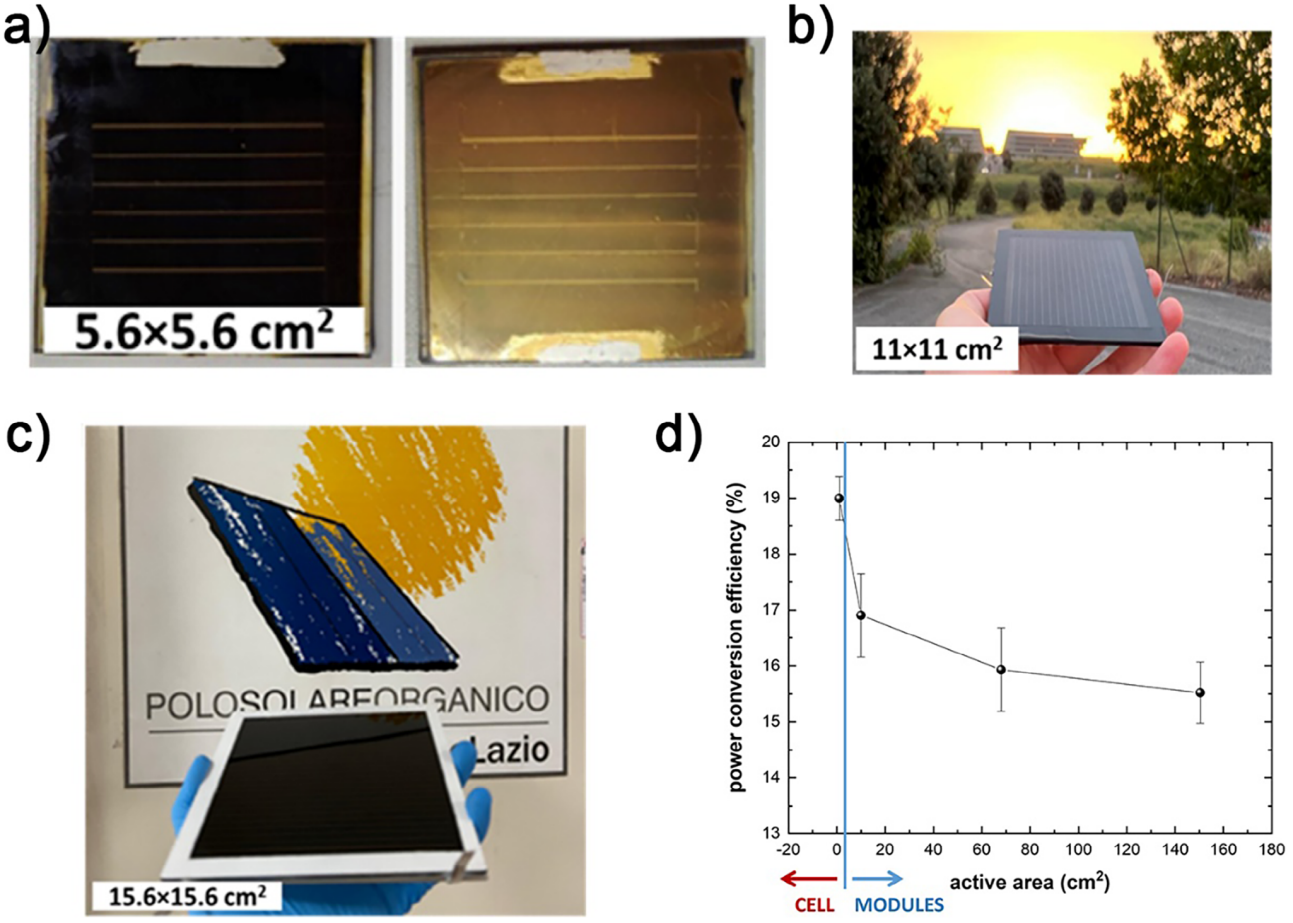
Photographs of representative PSMs with a) 5.6 × 5.6 cm^2^, b) 11 × 11 cm^2^ and, c) 15.6 × 15.6 cm^2^. d) Performance of PTAA‐based PSMs as a function of active areas, using the same cell configuration (layer stack). The PCE achieved at cell level (active area = 1 cm^2^) is also reported.

In this study, we detailed the upscaling process of PSMs with dimensions ranging from 5 × 5, to 15.6 × 15.6 cm^2^, with the aim of achieving high PCEs on both active (148 cm^2^) and aperture areas (156 cm^2^) for the largest modules (**Figure** **2**a–c). Further refinement in processing conditions is needed to ensure optimal performance at this scale. The optimized PSMs (15.6 × 15.6 cm^2^) were then integrated into large‐area (0.73 m^2^) PSPs, which were deployed within an existing solar farm in which various generation of PSPs developed by the authors in ref. [12] were tested, paving the way for practical and market‐competing perovskite‐based PVs. More in detail, PSMs were fabricated using an n‐i‐p structure in mesoscopic configurations. The m‐TiO₂ structure was selected for the n‐i‐p perovskite solar cell configuration over SnO₂‐based planar or p‐i‐n architectures for module fabrication due to its superior thickness (≈150 nm for c‐TiO₂/m‐TiO₂) compared to simple SnO₂. This increased thickness enhances device reproducibility, reducing potential issues such as shunts and other defects during scaling while ensuring greater stability and efficiency in n‐i‐p perovskite solar cells.^[^ ^26^ ^]^ The high surface area of its electron transport layer (ETL) enhances electron injection and reduces interfacial recombination, which is a critical limitation for obtaining efficient large‐area modules.^[^ ^12^ ^]^ Additionally, the high‐temperature sintering process ensures a robust and chemically stable ETL, enhancing long‐term stability. While SnO₂‐based planar structures require additional interface engineering for comparable stability, their fabrication methods often struggle with uniformity and scalability.^[^ ^27^, ^28^, ^29^ ^]^ Although atomic layer deposition (ALD) offers precise control, its slow growth rates and chamber size limitations hinder industrial‐scale production.^[^ ^30^, ^31^ ^]^ Moreover, mesoporous architectures support higher open‐circuit voltages (V_oc_) by minimizing trap‐assisted recombination, making mesoporous TiO₂ (mTiO₂) a preferred choice for high‐efficiency, scalable perovskite solar modules.

Building on our extensive experience,^[^ ^12^ ^]^ we incorporated single‐/few‐layer graphene flakes as electrically conductive dopants into the mTiO₂ based ETL, enhancing electron extraction toward the FTO electrode.^[^ ^32^ ^]^ Graphene flakes in compact TiO₂ (cTiO₂) reduced series resistance, improving the fill factor (FF),^[^ ^33^ ^]^ while their integration into (mTiO₂ regulated perovskite crystal growth,^[^ ^34^ ^]^ minimized charge trapping,^[^ ^35^ ^]^ and enhanced electron injection efficiency.^[^ ^36^ ^]^ Additionally, graphene flakes stabilized the perovskite/mTiO₂ interface,^[^ ^37^ ^]^ reducing charge thermalization and enabling hot‐carrier extraction architectures.^[^ ^38^ ^]^ Their presence also improved device stability by lowering trap state density,^[^ ^34^ ^]^ maintaining charge carrier temperature,^[^ ^38^ ^]^ and mitigating light‐induced perovskite degradation, which prevents gold and iodine diffusion across the structure.^[^ ^34^, ^37^, ^38^, ^39^ ^]^ Furthermore, we considered organic halide salt passivation, specifically phenylethylammonium iodide (PEAI), which fills iodine vacancies, reduces defects, and suppresses non‐radiative recombination.^[^ ^40^ ^]^ Upon annealing, PEAI forms a 2D PEA₂PbI₄ overlayer, enhancing structural stability and resistance to environmental degradation. These insights were incorporated into our study, validating the effectiveness of 2D material engineering in perovskite solar modules and panels.

Additionally, the cell interconnections were optimized through advanced laser patterning, increasing the GFF of the PSMs to 96.4%, thereby mitigating the performance gap between individual cells and the entire modules. Leveraging these advantages, our PSMs exhibited a PCE on active area as high as 17.68% and 16.32% with Spiro‐OMeTAD and poly(triarylamine) (PTAA), respectively, here selected as benchmark hole‐transporting layers for highly efficient PSCs. Remarkably, when scaling up from large‐area cells to modules, we achieved a PCE reduction of less than 19% despite increasing the dimensions by up to 150 times. For the PSMs based on Spiro‐OMeTAD as HTL, this reduction was even lower, at ≈8%, as shown in Figure 2d.

Multiple stability tests were conducted on PSMs, demonstrating their long‐term stability. We point out that only the PTAA‐based modules underwent lamination. In fact, when using Spiro‐OMeTAD as the hole transport layer (HTL), it is crucial to keep the lamination temperature below 120 °C to prevent material degradation and ensure device stability. While minimizing exposure time can mitigate thermal stress, the Spiro‐OMeTAD‐based module in our study was a non‐laminated prototype. In contrast, the large‐area laminated panel was composed of PTAA‐based modules, which provide enhanced structural robustness and allow for standard lamination conditions at 120 °C.^[^ ^12^, ^41^ ^]^ Subsequently, the PSMs were assembled and encapsulated through hot vacuum lamination of commercial thermoplastic foils, resulting in a 0.73 m^2^‐area PSP that achieved a PCE of up to 12.0%.

## 2 Perovskite Solar Modules and Panels Fabrication

### 2.1 Modules Fabrication

Mesoscopic n‐i‐p PSMs, each consisting of 24 cells with a 156 cm^2^ aperture area and a 150.4 cm^2^ active area (total area 243.4 cm^2^) were fabricated using the following layer stack: soda lime glass/fluorine doped tin oxide (FTO)/compact TiO_2_ + graphene (cTiO_2_ + G)/mesoporous TiO_2_ + graphene (mTiO_2_ + G)/triple cation perovskite (Cs_0.08_FA_0.80_MA_0.12_Pb(I_0.88_Br_0.12_)_3_)/PEAI/PTAA or Spiro‐OMeTAD/Au. Materials and methods used for the fabrication of PSMs are fully detailed in the Supplementary Information.

The high‐temperature deposition of cTiO_2_+G) via spray pyrolysis at 460 °C often leads to substrate cracking, particularly in larger modules. While smaller cells generally avoid this issue, scaling up to modules with dimensions exceeding 400 cm^2^ increases the risk of glass substrate breakage. Furthermore, processing large FTO glass at 460 °C can cause uneven bending, resulting in a height difference between the center and edges ^[^ ^42^ ^]^ an issue that is not commonly addressed in the literature. To overcome this issue, we refined the spray pyrolysis method by carefully modifying the heating ramp to prevent cracking of large‐area glass substrates.^[^ ^43^ ^]^ The primary causes for cracks during high‐temperature processes include uneven thermal expansion and contraction during annealing, as well as the presence of pre‐existing microcracks that act as stress concentration points during thermal cycling.^[^ ^44^ ^]^ We therefore made precise adjustments to the heating ramp and spray angle and performed sandpaper grinding to reduce the impact of micro‐cracks. These optimizations significantly lowered the breakage rate of glass substrates to less than 5%, compared to the standard process, which typically sees a breakage rate exceeding 85%. The optimized heating process, detailed in **Table** **1**, not only minimizes the costs by saving substrates but also accelerates the upscaling process. More in detail, we extended the ramp‐up time to ensure a gradual temperature increase, which helps prevent sudden tension on the substrates. Furthermore, we prolonged the duration of each step to maintain thermal stability throughout the process, thereby avoiding rapid expansion during heating and reducing the risk of substrate cracking.

**Table 1:** Optimized heating process parameters for spray pyrolysis.

Ramp up time (min)	5	10	20	20	60	60
Temperature (°C)	130	260	360	460	250	150
Steady temperature time (min)	5	5	15	30	5	–

Moreover, the proposed use of 2D materials for enabling perovskite panel production presents an exceptional and straightforward approach to tuning optoelectronic and chemical properties. The unique characteristics of 2D mat allow precise control over the perovskite module interface properties while enhancing the uniformity of material deposition.^[^ ^36^ ^]^

More in detail, incorporating graphene into the cTiO_2_ layer reduces dark current,^[^ ^33^, ^45^ ^]^ boosts the fill factor (FF),^[^ ^46^ ^]^ enhances long‐term PCE, mitigates UV‐induced photocatalytic degradation of functional materials and slows charge thermalization processes in perovskites.^[^ ^46^, ^47^, ^48^, ^49^ ^]^ Graphene flakes also facilitate the electron extraction from the perovskite to the anode ^[^ ^39^, ^45^ ^]^ and aid in regulating perovskite crystal growth, enhancing the reproducibility of the deposition process and minimizing defects in large‐area module fabrication.^[^ ^34^ ^]^ The 2D material interface engineering comprises not only the use of graphene in ELT but also PEAI to passivate the perovskite/HTL interface.^[^ ^50^, ^51^ ^]^ In dept, the crystallization of the perovskite film generates defects like vacancies and clusters that result in detectable pinholes and cracks, causing charge recombination, energy loss, and reduced open circuit voltage (*V*_oc_). These issues become more pronounced when depositing perovskite on large area.^[^ ^51^ ^]^ The PEAI passivation layer effectively fills these vacancies and surface boundaries, interacts with lead clusters, and improves film quality.^[^ ^52^ ^]^ Additionally, it provides a robust blocking layer that slows degradation from humidity or oxygen, inhibits ion migration, and imparts hydrophobicity.^[^ ^40^, ^50^ ^]^ For HTLs, we tested either PTAA or Spiro‐OMeTAD. Modules with Spiro‐OMeTAD exhibited higher PCE due to its superior hole mobility and film‐forming properties, which resulted in increased *V*_oc_ and short circuit current (*I*_sc_). However, PTAA presents significant advantages for scaling up and industrialization, thanks to its thermal and mechanical stability.^[^ ^53^ ^]^ Notably, PTAA‐based PSMs demonstrated enhanced thermal stability compared to Spiro‐OMeTAD, which tends to develop pinhole issues at elevated temperatures. Additionally, PTAA exhibits superior stability under ambient conditions, making it well‐suited for ensuring reproducible performance in PSMs, especially during the lamination process, which can reach temperatures of up to 120 °C.^[^ ^54^ ^]^

### 2.2 Laser Scribing Layout for High‐GFF Modules

The GFF is indeed a critical metric for assessing the performance of solar modules. It represents how effectively the module's surface area is utilized for active energy generation, with a higher GFF indicating that a larger portion of the module is devoted to capturing sunlight. A higher GFF means less wasted space, which, in turn, results in high PCE as well as cost‐effectiveness. The GFF value is defined as the ratio of the active area (W_a)_ to the total aperture area (W_a_ plus dead area, W_d_, also referred as inactive interconnection area), i.e.,^[^ ^55^, ^56^ ^]^:

(1) $$\mathrm{GFF}\;=\frac{Wa}{Wa+Wd}$$

**Figure 3:**
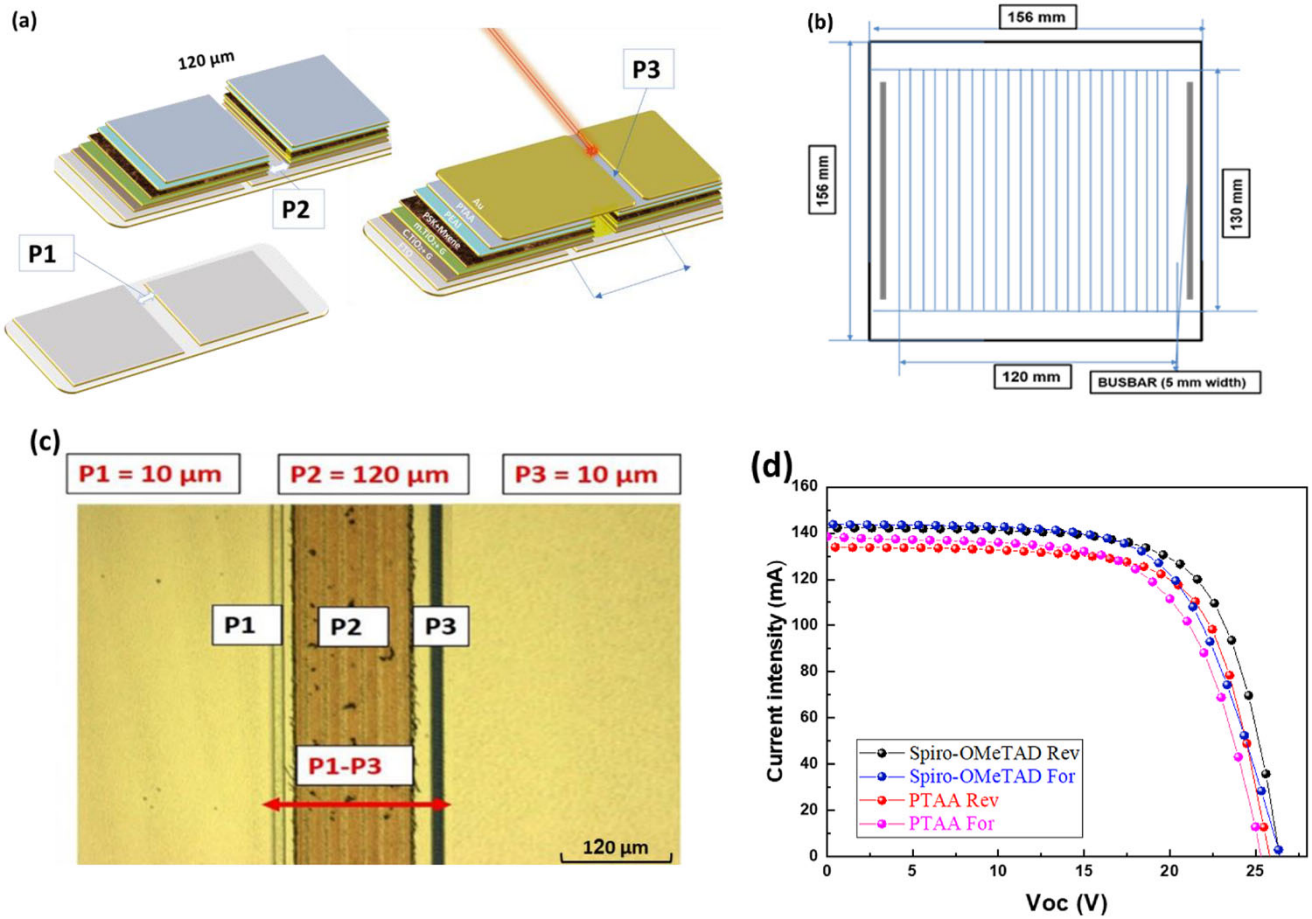
a) PSM laser layout for 15.6×15.6 cm^2^ with 24 sub‐cells. b) Schematic representation of P1, P2, and P3 widths. c) Laser ablation with minimized width for dead area, corresponding to a GFF of approximately 96.4%, d) *I–V* characteristics measured for the top‐performing modules with PTTA and Spiro‐OMeTAD as HTLs.

Traditionally, the fabrication of a monolithic series connection of cells composing a PSM involves three laser steps (P1, P2, P3), as depicted in **Figure** **3**a. However, the area between the P1 and P3 interconnection scribes remains inactive, resulting in area loss, which is quantified by the GFF.^[^ ^55^, ^56^, ^57^, ^58^, ^59^ ^]^ The laser layout design, shown in Figure 3b, features a rectangular configuration with a high length‐to‐width ratio (L/W). This design optimizes the cell geometry to maximize active area while minimizing total resistance of the cells. Compared to square‐shaped cells, this approach is more effective in optimizing the current flow and minimizing resistive losses, thereby improving the module's PCE.^[^ ^60^ ^]^ We utilized a 355 nm picosecond laser source for all the laser steps. The P1 step was executed on FTO substrates at a constant pulse density of 1200 pulses mm^−1^ with a pulse energy of 0.9 µJ and a speed of 150 mm ^−1^s, resulting in an ablation width of 10 µm. The P2 step stands out as the most extensively studied phase, requires precise optimization of laser parameters tailored to the specific device architecture and stack. This step entails the removal of the entire region of the heterogeneous stack that connects two adjacent cell stripes, except for the bottom FTO electrode. Residual TiO_2_ or damage to the TCO layer can severely degrade PSM performance. In particular, residual TiO_2_ can alter the interconnection contact between the metal back contact (Au) and FTO, changing it from an Ohmic contact to a Schottky junction.^[^ ^61^ ^]^

To ensure effective ablation on a module made of FTO/TiO₂ (both compact and mesoporous)/3D perovskite/PEAI/PTAA stack, we defined multiple etching lines with varying densities and power settings, as illustrated in Figure 3c. Specifically, 6 parallel scribing lines were used for the P2 process, each with different pulse density and ensuring thorough ablation. This set of lines determines the Raster Scanning Distance (RSD) value. This value can affect the recombination rate at interconnection interfaces, subsequently affecting the series resistance and FF in PSMs.^[^ ^56^, ^62^ ^]^ For the P2 step, a 10 µm RSD was used with density of 1200 pulses m^−1^ at a pulse energy of 0.6 µJ, resulting in a narrow width dead area of ≈120 µm. The ablation power was sufficient to eliminate the entire heterogeneous stack from the FTO. The sheet resistance of the ablated area, measured using four‐probes Keithley 2400 system, was 8 Ω/◻, closely matching the sheet resistance of bare FTO. Finally, in the P3 step, the Au electrode was ablated to electrically isolate adjacent cells by horizontally separating the top metal contacts and charge transport layers, using shallow scribing with the same laser system. This step was performed with a pulse energy of 0.1 µJ with a pulse density of 1000 pulses/m, resulting in a µm‐width ablation area. The so‐designed PSMs, fabricated on 15.6 × 15.6 cm^2^ substrate, consist of 24 series‐connected cells, each with a 0.5 cm cell width, a 156 cm^2^ aperture area and a 150.4 cm^2^ active area. The P1‐P3 interconnection distance was maintained at 180 µm, resulting in a dead area per cell of 23.4×10⁻^2^ cm^2^, which is less than 4% of the aperture area. This results in a GFF of 96.4% (Table S1, Supporting Information), the highest values reported for PSMs of this size. Only two other studies have reported higher GFF values, both for smaller devices. The highest reported GFF for smaller devices, with an area of 2.62 cm^2^, is ≈99.6%.^[^ ^63^ ^]^ Our accomplishment demonstrates a highly efficient utilization of the module's surface, maximizing light absorption and, consequently, enhancing overall PCE.

### 2.3 PSMs Performance

The PSMs were fabricated using a FTO/CTiO_2_/mTiO_2_/triple cation perovskite (Cs_0.08_FA_0.80_MA_0.12_Pb(I_0.88_Br_0.12_)_3_))/PEAI/PTAA/Au stack, selected for its superior stability compared to other configuration, as reported by our group ^[^ ^51^, ^64^ ^]^ and other literature.^[^ ^49^, ^65^ ^]^ To facilitate the scaling up, we reproducibly fabricated PSMs of dimensions 2.5 × 2.5 cm^2^, 5.6 × 5.6 cm^2^ (Figure S1, Supporting Information), 11 × 11 cm^2^ (Figure S2, Supporting Information), and 15.6 × 15.6 cm^2^, aiming to maintain optimal performance across different sizes using the same stack configuration also optimizing the laser parameters (further detailed in Figure S3 and paragraph Methods, Supporting Information). In particular, we reached a PCE of 17.94% for the 5.6 × 5.6 cm^2^ PSMs, which represents only an 8% PCE loss compared to large‐area cells. As we further scale up, we achieved a PCE of 17.21% for 11 × 11 cm^2^ PSMs, indicating a performance decrease of less than 4% compared to the previous PSM size (**Table** **2**, Figure 2e). Moreover, the FF decreases with increasing active area due to the higher number of interconnected cells, which raises series resistance. Additionally, larger cell dimensions may intensify defects from the P2 ablation process. Since FF directly influences PCE, its trend also impacts on the overall PCE performance. (see Figure S4, Supporting Information)

**Table 2:** Photovoltaic characteristics of PSMs with same stack for different dimensions (from 6.25 – 243.4 cm^2^).

Substrate dimension [cm^2^]	Aperture/active area [cm^2^]	*V*_oc_[V]	*I*_sc_ [mA]	FF [%]	PCE [%] on active area
2.5 × 2.5	1/‐	1.11	23.88	74.78	19.45
5.6 × 5.6	11/10	5.46	42.67	76.94	17.94
11 × 11	70/68	19.17	87.63	65.59	17.21
15.6 × 15.6	156/150.4	25.50	133.30	70.1	16.32

We fabricated 90 PSMs with dimensions of 15.6 × 15.6 cm^2^, utilizing a mesoporous structure. For this dimension, the I‐V curves for the top‐performing PSMs are shown in Figure 3d. The stabilized best‐PCE of these modules was equal to 16.32% and 17.68% when using PTAA and Spiro‐OMeTAD HTLs, respectively (**Table** **3**). The PCE of 15.6 × 15.6 cm^2^ PTAA‐based PSM was 5%, lower than that of the 5.6 × 5.6 cm^2^ PSM, and showed a reduction of less than 19% compared to large‐area cell with 1 cm^2^ active area. Moreover, PTAA‐based PSMs have 8% lower PCE than Spiro‐OMeTAD PSMs, as indicated in Table 3.

**Table 3:** Performance Comparison of 15.6 × 15.6 cm^2^ PSMs with different HTLs.

Module	HTL	*V*_oc_[V]	*I*_sc_ [mA]	FF [%]	PCE [%]
1	PTAA	25.5	133.3	70.1	16.32
2	Spiro‐OMeTAD	26.6	141.8	69.3	17.68

**Figure 4:**
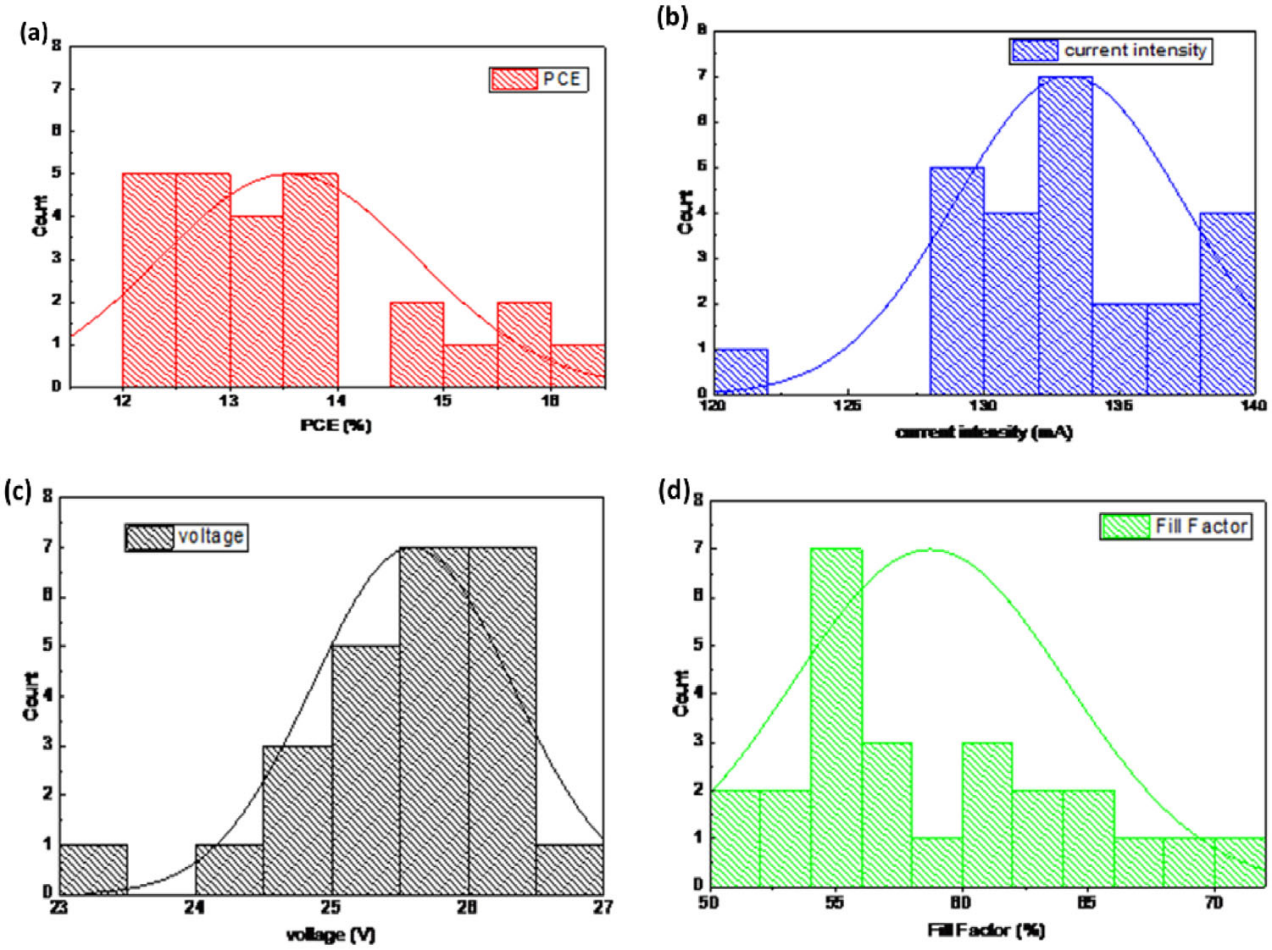
a) Statistical analysis of the photovoltaic characteristics measured for the investigated PSMs: a) PCE, b) *I*_sc_, c) *V*_oc_, d) FF.

The statistical analyses of the PV parameters measured for 25 PSMs, used to assemble the 0.73 m^2^ PSP, are depicted in **Figure** **4**a–d. The average values for PCE, *V*_oc_, (absolute), current and FF are 13.6 ± 1.2%, 25.6 ± 0.7 V, 133.1 ± 4.2 mA, and FF of 58.7 ± 5.4%, respectively. The performance variations and standard deviations are related to the duration and complexity of the fabrication process for the PSMs over a period of 5 months, as detailed hereafter. Before PSP assembly, the stability of PSMs was assessed by proper encapsulation. The latter was performed through a hot vacuum lamination method, laminating an aluminum/thermoplastic ionomer (Jurasol) foil system atop the module rear contact foil.^[^ ^66^, ^67^ ^]^ We selected ionomer‐based barrier foils for their rapid curing, resistance to discoloration, and reduced risk of degradation compared to traditional EVA. Unlike EVA, the ionomer‐based material does not release corrosive acetic acid, making it particularly suitable for emerging PV technologies like perovskite‐based ones.^[^ ^68^ ^]^ The PCE loss observed after the encapsulation of PTAA‐based PSMs is attributed to both thermal stress during the lamination process ^[^ ^53^ ^]^ and mechanical stress originated by the mismatch of thermal expansion coefficients of the materials composing the cell stack ^[^ ^69^ ^]^ Prospectively, advanced encapsulant, as viscoelastic semi‐solid adhesives recently developed at lab‐scale from our groups, can reduce the thermomechanical stresses at the different layer interfaces.^[^ ^70^ ^]^ The thermal stability of the encapsulated PSMs was tested according to the ISOS‐D2 protocol (dry heat at 85 °C). A T80 of 1250 h was measured (Figure S5, Supporting Information) for the PTAA‐based PSM.

### 2.4 Lamination Process for the Panel

**Figure 5:**
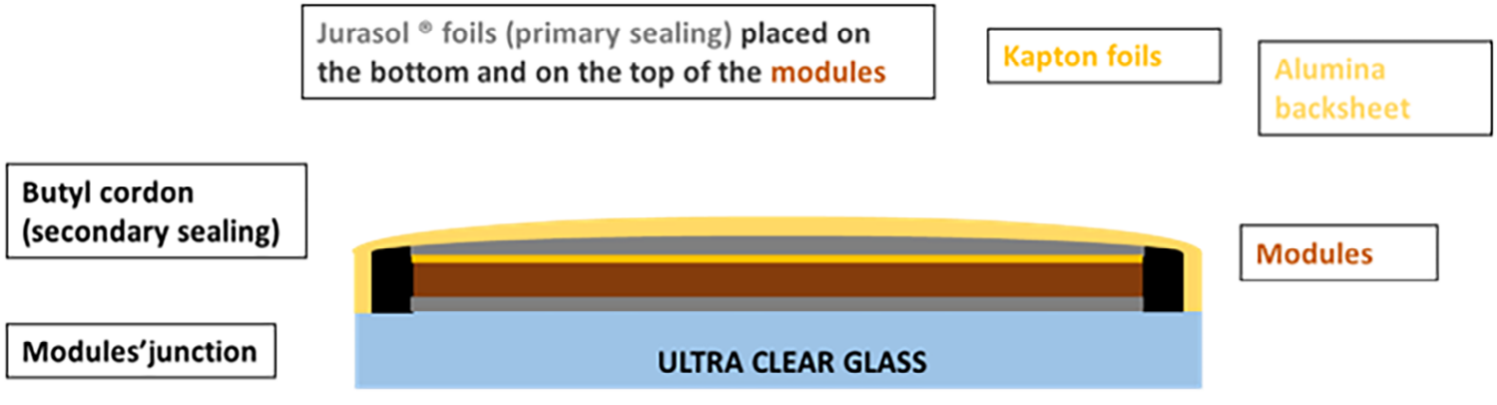
Side‐view panel stack encapsulated by hot vacuum lamination of thermoplastic ionomer (Jurasol^TM^) at 120 °C, showcasing the two PSM raws.

The encapsulation process plays a pivotal role for achieving long‐term stability of highly performing PSPs. By sealing the sensitive perovskite layer between protective materials, encapsulation improves resistance to environmental factors such as moisture, oxygen, and UV radiation, which can otherwise degrade functional materials over time. Additionally, encapsulation also maintains the structural integrity of the panel, preventing module interconnection failures and/or damage during handling and deployment into solar farms. Even more, encapsulation can also boost the PCE by reducing light reflection and enhancing light trapping within the device.^[^ ^71^, ^72^, ^73^ ^]^ As illustrated in **Figure** **5**, the encapsulation of a 0.73 m^2^ PSP was performed through hot a vacuum lamination using a foil of commercial thermoplastic ionomer (Jurasol),^[^ ^66^, ^67^, ^68^ ^]^ following the protocols reported for PSMs.

**Figure 6:**
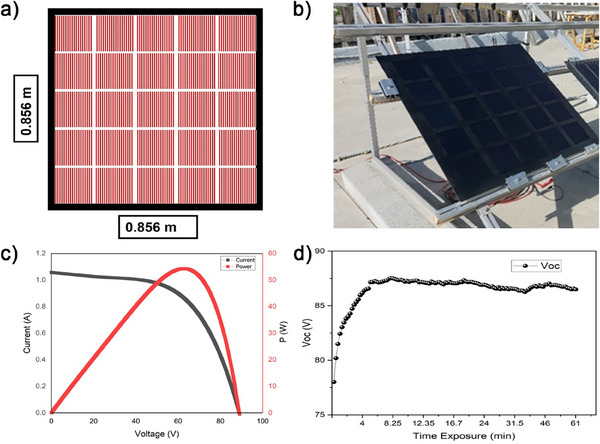
a) Schematic top‐view of PSP configuration, consisting of 5 raw connected in parallel, with each raw made of 5 PSMs connected in series. b) Photograph of the panel installed at the HMU@nano solar field in Crete for outdoor characterization. c) *I–V* curve measured for the PSP after lamination. The power is also plotted as a function of the voltage, d) *V*_oc_ measured for our PSP under 1 sun illumination at MPP tracking over time.

The entire PSP manufacturing chain is depicted in Figure S6 (Supporting Information). Specifically, PSMs were connected in a series within each row, and the rows were subsequently connected in parallel. Modules within each row were selected based on current matching, while the rows were configured to ensure a similar voltage output. This configuration optimizes the overall performance of the PSP and minimizes mismatches between the currents and voltages of the modules. The arrangement of the PSMs within the PSP is depicted in **Figure** **6**a, while the photo of the panel installed at the HMU@nano solar field is reported in Figure 6b. The front side of the panel consists of 4 mm thick tempered glass, serving as the foundation for the lamination process. A thermoplastic sheet is first placed on the glass, followed by the modules, which are then sandwiched between additional thermoplastic layers. To prevent delamination of the back electrode during hot vacuum lamination, which could lead to the migration of gold fragments causing shorts between adjacent cells ^[^ ^62^ ^]^ a Kapton sheet was added between the back electrode side of the modules and the second thermoplastic sheet. HelioSeal tape was applied along the frame of the panel to seal out moisture and oxygen while also reinforcing the lamination structure. Lastly, a multilayer back‐sheet foil (PA‐PET‐ALU‐PA) was used as a back cover to provide additional support to the connected modules, as shown in Figure 5. Despite the main focus of this study is the upscaling and performance of perovskite solar modules (PSMs) and panels (PSPs), the impact of thermocompression encapsulation on long‐term stability and performance should be considered. In fact, thermal and mechanical stresses introduced during encapsulation employing high‐temperature processes, such as vacuum lamination at 150 °C, can accelerate ion migration, phase segregation, and interfacial degradation, leading to performance losses.^[^ ^74^ ^]^Additionally, mechanical stresses arising from the mismatch in thermal expansion coefficients between encapsulant materials and perovskite layers can lead to interfacial degradation.^[^ ^70^ ^]^ Furthermore, photoinduced halide segregation can deteriorate the film surface morphology, forming nanoscale cracks that exacerbate performance losses.^[^ ^75^ ^]^ To address these issues, alternative encapsulants, such as strain‐free viscoelastic adhesives with thermally conductive fillers, have shown promise in reducing stress, improving heat dissipation, and enhancing moisture protection.^[^ ^70^ ^]^ However, none of these proposed solutions are currently available on an industrial scale. Therefore, in this work, we utilized standard encapsulants while minimizing the occurrence of mechanical, thermal, and degradation stresses by reducing both the lamination temperature and processing time. Future research needs to be explored to employ next generation encapsulant materials and methods to further mitigate degradation effects and enhancing the durability and performance of perovskite solar devices. Finally, the panel was tested outdoors at the HMU@nano solar field in Crete, where we have already installed a solar farm built with perovskite‐based PSPs.^[^ ^12^ ^]^

The *I–V* characteristic of our PSP after lamination shows a PCE on active area of 12.0 ± 1.6% and a maximum power of 65 W, as shown in Figure 6c. Furthermore, Figure 6d shows the *V*_oc_ as a function of time under MPP tracking under 1 sun illumination in outdoor conditions. The fluctuation in *V*_oc_ observed in Figure 6d is due to variations in environmental conditions during the outdoor measurement. Factors such as temperature fluctuations, changes in solar irradiance, and minor shading effects can contribute to small variations in *V*_oc_ over time. (see Figure S7 and paragraph Outdoor measurement in Supporting Information for further details) Additionally, the stabilization process of the PSP under continuous operation can also cause a gradual increase in V_oc_. Despite these minor variations, the *V*_oc_ stabilizes ≈87 V, demonstrating the reliable performance of the device under MPP tracking.

**Figure 7:**
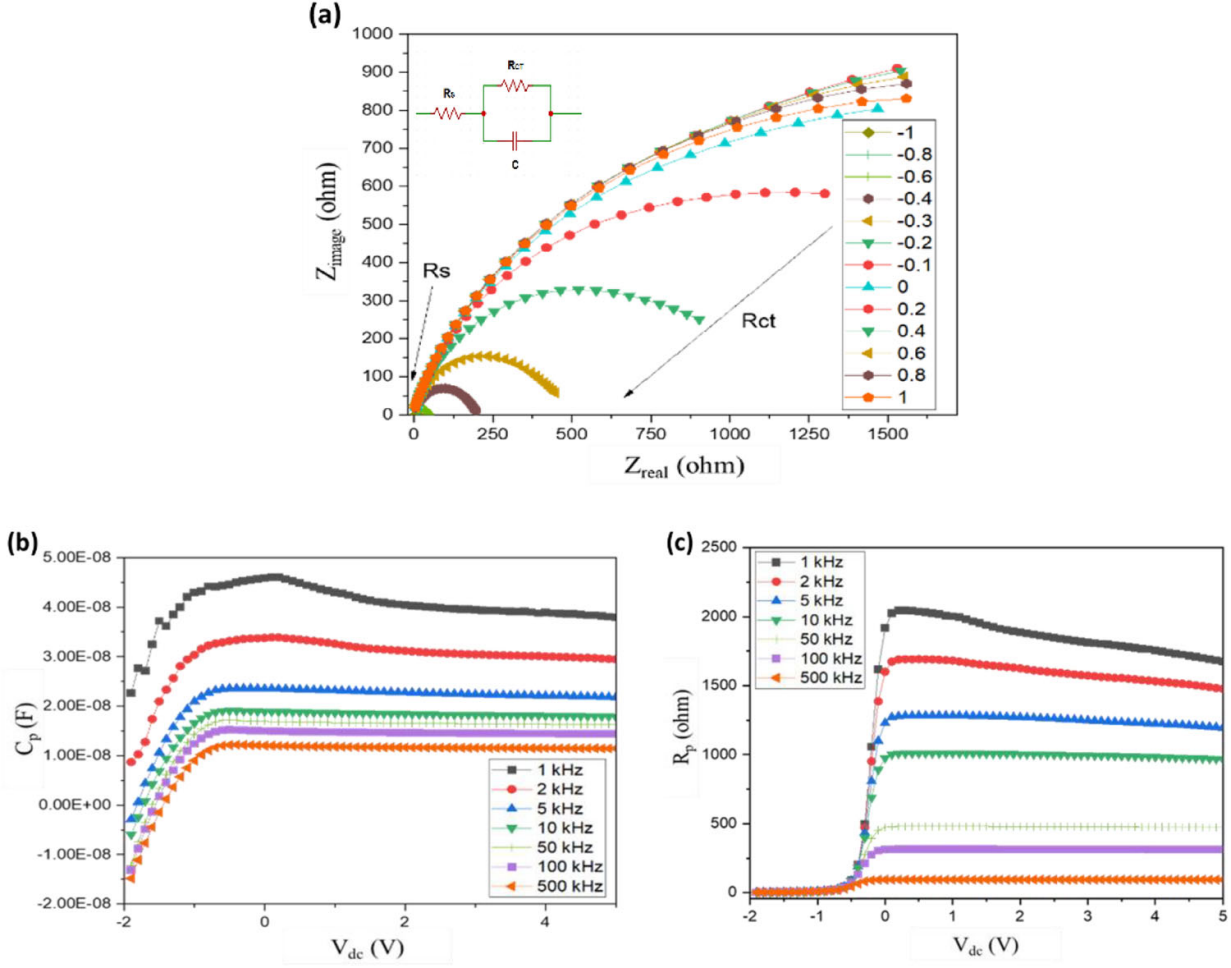
a) EIS analysis of the PSP, showing Nyquist plots measured at various *V*_bias_, ranging from ‐1 V to 1 V. The equivalent electric circuit of the system is based on a series resistance (R_S_) and a charge transfer resistance (*R*_CT_) connected in parallel with a capacitor. b) Apparent capacitance (C_p_) of the PSP, calculated as Im(1/Z)/ω, and c) polarized resistance (R_p_) plotted as a function of DC voltage and frequency, ranging from 1 kHz to 500 kHz.

Electrochemical impedance spectroscopy (EIS) measurements were performed on our PSP to assess its interfacial charge transport dynamics across a frequency range of 1 kHz to 500 kHz and DC voltage biases (*V*_bias_) from −1 to 1 V The equivalent circuit model of perovskite panel as an entity consists of a combination of the external series resistance and a charge transfer resistor connected in parallel with a capacitor (inset in **Figure** **7**a). R_s_ (series resistance) includes contacts resistance, wire resistance, sheet resistance of the electrode and polarized resistance of the module. The Charge transfer resistance, *R*_CT_, is associated with the charge transport resistance of the bulk perovskite and is also influenced by the transport resistance of the ETL.^[^ ^76^ ^]^The capacitor C, represents the nonideal geometric capacitance and is associated with the dielectric response of the perovskite layer in high‐frequency region (>1 kHz) of the spectra. The EIS characteristics show that in high frequency the polarized resistance (R_p_) and C values decrease, related to the dielectric properties of the perovskite material. *R*_CT_ decreased in low dc applied voltage, associated with an increase of leakage current and recombination processes.^[^ ^77^ ^]^ As shown in Figure 7a, when *V*_bias_ are more negative than −0.4 V, the panel's bypass diodes are activated, permitting a reverse current flow. This activation is evident in the reduction of R_CT_, as indicated by the decreasing arc radius in the Nyquist plots. Separate Nyquist plots for negative and positive *V*_bias_ are presented in Figure S8 (Supporting Information) for comparison. Figure 7b illustrates that apparent capacitance (C_p_) increases as frequency decreases. Specifically, when *V*_bias_ is −0.3 V (brown curve in Figure 7a), the semicircle radius decreases and nearly reaches to the real part of the impedance (*Z*_real_) axis. Additionally, Figure 7c shows the resistance (R_p_) of the PSP across various V_dc_ as a function of the frequency. The PSP exhibits higher R_p_ at lower frequencies which is likely due to charge carrier recombination and slower ionic movements.^[^ ^77^ ^]^ Further insights into the system's transfer function behavior, denoted as G(S) in the frequency domain, are available in supplementary information (see Figure S9, Supporting Information). Thermal imaging and heat distribution analysis, performed using an infrared camera during field testing, indicated that temperatures in some areas of the panel can reach up to 59.3 °C, as shown in Figure S10 (Supporting Information). This suggests localized overheating, which could affect the panel's performance and stability.

We initially aimed to assess the stability of the PSP under outdoor conditions; however, the testing was cut short due to PSP failure, exposing significant challenges related to the panel's encapsulation process. The use of thermoplastic ionomers (Jurasol in this case) as an encapsulant proved problematic, likely due to their stiffness. This rigidity can cause cell delamination during thermomechanical stress which occurs both during encapsulant application though lamination and during day/night temperature cycle. In particular, the Young's modulus of thermoplastic ionomers is typically higher than that of other encapsulants, including ethylene vinyl acetate (EVA) ^[^ ^78^ ^]^ that was previously used for building our first perovskite‐based solar farm described in ref.[12] Generally, encapsulants with a low Young's modulus are preferred for PSCs and PSMs to prevent delamination, especially given the mismatch in thermal expansion coefficients between different materials in the module and panel stacks. Noteworthy, in this work, we introduced a Kapton foil ^[^ ^12^ ^]^ between the encapsulant foil and the PSM, which means that we rationally avoided the risk of module structure delamination during the module encapsulation with the ionomer. Thus, the PSP failure was associated with failures in the electrical connections between PSMs as well as to the presence of air pockets between the Kapton and PSMs, impeding a tight seal. The absence of a tight seal cannot constrain the outgassing of volatile species, which is mandatory for high‐stability PSCs.^[^ ^79^ ^]^ Air pockets can act as sites for thermal and mechanical stress concentration. During temperature fluctuations, the differential expansion of materials can cause these pockets to expand or shift, potentially leading to further mechanical damage, such as cracking or delamination of the encapsulant from the underlying layers. This not only exacerbates the ingress of environmental contaminants but also contributes to the loss of electrical continuity and increased resistance at the interconnections, thereby reducing the overall efficiency and stability of the solar panel.

## 3 Conclusions

In this paper, we have demonstrated the successful scalability of perovskite solar cell (PSC) technology from laboratory to industrial‐scale applications, achieving significant power conversion efficiencies (PCEs) of 16.32% and 17.68% for perovskite solar modules (PSMs) using PTAA and Spiro‐OMeTAD as hole transport layers, respectively. Our findings push the boundaries of PCE for modules, setting new standards for industrial feasibility. Importantly, we achieved a geometric fill factor (GFF) of 96.4% for a PSM with an aperture area of 156 cm^2^, a notable milestone for this scale.

Real‐world testing is essential for any photovoltaic (PV) technology, so our PSMs were used to fabricate a 0.73 m^2^ perovskite solar panels (PSP). The latter was subjected to field testing within an operational solar farm, achieving a remarkable PCE of 12.0 ± 1.6%, making it the largest PSP reported in scientific literature results for this specific dimension. This real‐world performance underscores the practicality and promise of our approach in solar energy generation. However, the study also reveals challenges associated with using high‐stiffness encapsulants, which may not be ideal for thin‐film solar cells. Looking forward, advanced encapsulants offer promising solutions to these challenges. We have recently demonstrated the potential of semi‐solid encapsulants at the lab scale,^[^ ^70^ ^]^ and our future work will focus on upscaling reliable blanket‐cover encapsulation methods to ensure stable perovskite‐based PVs at the panel level. Moreover, we have already explored the possibility of replacing the gold counter‐electrode with a graphene‐based carbon paste.^[^ ^80^ ^]^ This, together with the scaling‐up results demonstrated in this work and the blanket approach encapsulation method, drives the industrial advancement of 2D‐based perovskite photovoltaic technology, further pushing the boundaries of emerging PV innovation.

## Conflict of Interest

The authors declare no conflict of interest.

## Supporting information



Supporting Information

## Data Availability

The data that support the findings of this study are available from the corresponding author upon reasonable request.
