# Supplementary material for: Upscaling Perovskite Photovoltaics: from 156 cm2 Modules to 0.73 M2 Panels
Source: Adv Sci (Weinh). 2025 Apr 26;12(22):2416316. doi: 10.1002/advs.202416316 (PMC12165116; doi:10.1002/advs.202416316)
Supplement: Supplementary file 1 [file ADVS-12-2416316-s001.odt]

SUPPORTING INFORMATION

Upscaling Perovskite Photovoltaics: From 156 cm2 Modules to 0.73 m² Panels.

Hafez Nikbakht,1 Paolo Mariani,1 Luigi Vesce1, Francesco Di Giacomo1,2, Enrico Leonardi,3 George Viskadouros,4,5 Emmanuel Spiliarotis4, Konstantinos Rogdakis4,6, Sara Pescetelli1, Antonio Agresti1, Sebastiano Bellani7, Francesco Bonaccorso7,*, Emmanuel Kymakis,4, 6,* and Aldo Di Carlo1,8,*

- CHOSE – Centre for Hybrid and Organic Solar Energy, University of Rome Tor Vergata, via del Politecnico1, 00133 Rome (Italy)

- Solertix via Eusebio Chini 15, Rome 00147, Italy

- GreatCell Solar Italia SRL, viale Castro Pretorio 122, 00185 Rome, Italy

- Department of Electrical & Computer Engineering, Hellenic Mediterranean University, Heraklion, GR71410, Crete, Greece

- Department of Mineral Resources Engineering, Technical University of Crete, Chania 731 00, Crete, Greece

- Institute of Emerging Technologies, University Research and Innovation Center, HMU, Heraklion 71410, Crete, Greece

- BeDimensional S.p.A., Via Lungotorrente Secca, 30R, Genova, 16163 Italy

- Istituto di Struttura della Materia-Consiglio Nazionale delle Ricerche Roma (CNR-ISM), via del Fosso del Cavaliere 100,Rome00133, Italy

* Corresponding author. [aldo.dicarlo@uniroma2.it](mailto:aldo.dicarlo@uniroma2.it); [kymakis@hmu.gr](mailto:kymakis@hmu.gr); [f.bonaccorso@bedimensional.it](mailto:f.bonaccorso@bedimensional.it)

Materials

TiO2 (titanium dioxide) paste (30 NR-D), formamidinium iodide (FAI), methylammoniumbromide (MABr), were purchased from GreatCell Solar. Lead(II) iodide (PbI2), lead(II) bromide (PbBr2), and cesium iodide (CsI) were purchased from TCI. Cesium bromide beads (CsBr), titanium(IV) isopropoxide (TTIP), diisopropoxytitanium bis(acetylacetonate) (Ti(AcAc)2), acetyl acetone (AcAc), phenethylammonium iodide (PEAI), tris(2-(1H-pyrazol-1-yl)-4- tert-butylpyridine)cobalt(III), tri[bis(trifluoromethane)sulfonimide] (FK209 Co(III) TFSI), ethanol (EtOH) (anhydrous, ≥99.8 %), acetone (≥99.5%), acetonitrile (ACN) (≥99.8%), dimethylformamide (DMF) ≥ 99%), dimethyl sulfoxide (DMSO) (>99%), chlorobenzene (CB) (99.8%), 1,2-dichlorobenzene (DCB) (99%), toluene (>99.7%), N‐methyl‐2‐pyrrolidone (NMP) (>97%), 2-propanol (IPA) (anhydrous, 99.5%), ethyl acetate (anhydrous, 99.8%), tBP and LiTFSI were purchased from Sigma-Aldrich. Poly[bis(4-phenyl) (2,4,6-trimethylphenyl)amine] (PTAA) SOL2426M (average molecular weight, 105 kDa) was purchased from Solaris Chem. [6,6]-phenyl-C61-butyric acid methyl ester was purchased from Solenne. Graphene dispersion in EtOH (0.9mg/mL) was supplied by BeDimensional S.p.A. Room temperature highly viscous (Brookfield viscosity >100000 mPas at 10 rpm at temperature <120 °C). All the chemicals were used as received unless specified otherwise. Fluorine Tin Oxide (FTO)-coated glasses (sheet resistance RSH=7 Ω/sq) were purchased from NSG-Pilkington. Silver (Ag) paste 7713 was purchased from Dupont. 

Methods

Mesoscopic PSCs (active area = 1 cm2) were fabricated on SLG/FTO-coated SLG glass substrates cut in 2.5 × 2.5 cm2 size. The FTO layer was patterned via laser etching using an Nd:YVO4-pulsed UV laser system (BrightSolutions, Luce 40 laser), carrying out a P1 process to electrically separate the photoelectrode from the counter electrode.

After the etching step, the substrates were cleaned with brushing and without scratching the FTO surface, using a cleaning solution (Hellmanex) diluted with water (2:98 vol/vol). After this step, the substrates were sonicated in an ultrasonic bath first with acetone and then with IPA (10min for each step). Afterwards, the substrates were air dried. Then, an UV/O3-treated with a PSD Pro Series Digital UV Ozone System (Novascan) was used to remove organic contaminations. The c-TiO2 layers were then deposited onto the patterned FTO by the spray pyrolysis of a dispersion of 0.16 M Ti(AcAc)2 and 0.4M AcAc in EtOH, setting the hot plate temperature at 465°C and using air as the gas carrier at a pressure of 1.6 bar. The nozzle angle was about 45°C with respect to the plane of the substrate, and the nozzle was moved with a serpentine path for 12-13 cycles (one every 10 seconds) until reaching a thickness of 50 nm, as measured by profilometry (Deektat Veeco 150). Then, the substrates were left for 15min at 465°C before slowly cooling them to room temperature. To fabricate them-TiO2 layers, the 30NR-D TiO2 paste was diluted in anhydrous EtOH (1:5 w/w). After stirring overnight (>12 h), graphene was incorporated into the diluted 30NR-D TiO2 paste by adding 1 vol% graphene dispersion in EtOH. 120 μL of the graphene-incorporating TiO2 diluted paste was spin coated onto c-TiO2 layers at 3000 rpm for 30 s with an acceleration of 1500 rpm/s. The resulting layers were converted into graphene incorporating m-TiO2 through a multi-step sintering program, with the same steps described in the main text for the spray pyrolysis excepted for the fourth step, in which T has been set to T = 485°C. To improve its wettability, the graphene-incorporating m-TiO2 layer was UV-treated for 30min with 5000-EC UV curing lamps (Dymax). Then, the samples were transferred to the glove box. The perovskite precursor solution was then prepared by dissolving FAI (1M), PbI2 (1.2M), PbBr2 (0.2M), CsI (0.1M), MABr (0.2M) in DMF and DMSO (1:4 vol/vol) to obtain a perovskite composition of Cs0.08FA0.80 MA0.12Pb(I0.88Br0.12)3. After stirring overnight, 90 μL of the perovskite precursor solution was spin-coated on the ETLs with a two-step protocol: 1) 2000 rpm for 10 s with an acceleration of 400 rpm/s; 2) 5000 rpm for 20 s with an acceleration of 2000 rpm/s. Subsequently, 100μ L of CB, used as antisolvent, was poured on the spinning substrates 15 s before the end of the spinning programme to induce the perovskite nucleation. After the perovskite deposition, the samples were annealed at 100°C for 45 min to complete the crystal phase growth. To ensure the reproducibility and high quality of our perovskite films, we maintain a constant processing temperature inside the glovebox at around 22-23°C, minimizing variations that could arise from external environmental fluctuations. This controlled atmosphere allows us to optimize the crystallization dynamics and morphological uniformity of the perovskite layer, ensuring consistent device performance.

An ultrathin perovskite passivating PEAI layer was then deposited onto the perovskites by spin coating 100 μL of EI solution in IPA (concentration of 5mg/mL) at 2500 rpm for 30 s, with an acceleration of 1250 rpms. Then, the samples were annealed at 100°C for 10 min. To deposit the hole transport layer, a PTAA solution in toluene (10 mg/mL), doped with 7 μL/mL of tBP and a 10 μL/mL of a LiTFSI stock solution in ACN (170 mg/mL), was spincoated at 3000 rpm for 20 s with an acceleration of 1500 rpm/s. As alternative to PTAA, spiro-OMeTAD was deposited by spinning 90 μL of spiro-OMeTAD solution in CB (73.5 mg/mL) doped with tBP (26.8 μL/mL), LiTFSI (16.6 μL/mL) (from stock solution in ACN (520mg/mL) and FK209 Co(III) TFSI (7.2 μL/mL), at 4000 rpm for 1min, with an acceleration of 1000rpm/s. Finally, themesoscopic n-i-p PSCs were completed by depositing 100 nm-thick Au back electrodes through thermal evaporation in a high vacuum chamber (10−6 mbar).

Mesoscopic PSMs (active area = 10 cm2) were fabricated onto FTO-coated glasses with sizes of 5.6 cm x 5.6 cm. These substrates were patterned with a P1 process, defining the active area (10 cm2) distributed in 5 cells, each of them with an area equal to 2 cm2. The distance between one P1 line and the sequent one was 5.5 mm. The cell length was 40 mm. The fabrication steps were the same for the mesoscopic PSCs, except for the amounts of solutions dropped onto the substrates. The amounts of m-TiO2 solution, perovskite precursor solution, chlorobenzene as antisolvent, PEAI solution, and PTAA solutions were 600 μL, 450 μL, 400 μL, 450 μL and 500 μL, respectively, for each module. The sintering process was carried out in an oven, Lenton WHT6/60 (Hope Valley, U.K.) with the same ramp described for the cells. P2 and P3 process are the same described in the main text. By using a laser, we obtained a width of P2 equal to 160 μm. After the thermal evaporation of the 100 nm-thick Au back electrodes, the adjacent cell isolation was accomplished with the P3 process. The width of P3 was 90 μm. The resolution of our laser ablation processes led to a geometrical fill factor (GFF) of ~91%.

 

Figure S1. Layout of the 10 cm2 PSMs.

Mesoscopic PSMs (active area = 68 cm2) were fabricated onto FTO-coated glasses with sizes of 11.0 cm x 11.0 cm. These substrates were patterned with a P1 process, defining the active area (64 cm2) distributed in 17 cells, each of them with an area equal to 4 cm2. The distance between one P1 line and the sequent one was 5.5 mm. The cell length was 80 mm. The fabrication steps were the same for the mesoscopic PSCs, except for the amounts of solutions dropped onto the substrates. The amounts of m-TiO2 solution, perovskite precursor solution, chlorobenzene as antisolvent, PEAI solution, and PTAA solutions were 1 mL, 950 µL, 1 mL, 1.0 mL and 1.0 mL, respectively, for each module. The sintering process was carried out in an oven, Lenton WHT6/60 (Hope Valley, U.K.) with the same ramp described for the cells. By using a laser, we obtained a width of P2 equal to 180 μm. After the thermal evaporation of the 100 nm-thick Au back electrodes, the adjacent cell isolation was accomplished with the P3 process. The width of P3 was 150 μm. The resolution of our laser ablation processes led to a GFF of ~92%.

Figure S2. Image at confocal microscope of the P1-P2-P3 process on the 64 cm2 active area modules.

To assess the impact of the P2 process on final module performance, we optimized a P2 process for removing the perovskite-based cell stack from the interconnection area between adjacent cells. After depositing the cell layers, a second laser etching step, called the P2 process, is required to clean the FCO area, which hosts the monolithically integrated vertical interconnections typically deposited via thermal evaporation when creating the counter electrode (CE). The P2 etching process must be strong enough to effectively remove the cell layers and thoroughly clean the FCO contact while being gentle enough to avoid damaging the FCO, which could increase series resistance and reduce module performance. Additionally, the P2 ablation process causes significant degradation in the adjacent active zone. Therefore, the P2 ablation parameters—laser fluence, pulse repetition rate (PRR), and raster scanning distance (RSD)—must be optimized to ensure quick and efficient FCO cleaning while minimizing degradation of the cell’s active area. To this end, we define a parameter matrix with different power densities and frequencies to systematically evaluate the ablation quality. Based on the best-performing combination, we select the most suitable parameters to achieve uniform and complete ablation. Our process involves six distinct laser scribes, grouped into three pairs with different parameter settings, ensuring that each stage contributes to effective material removal. The first two scribes operate at a higher pulse density to ensure aggressive ablation and efficient material removal. The intermediate scribes balance ablation depth and thermal diffusion, while the final two scribes, near the P3 region, utilize lower power densities to refine the edges and prevent excessive damage to the underlying layers. This structured approach ensures full ablation of the P2 region across the entire module, even in the presence of thickness variations.

The presence of residuals in the P2 region can be attributed to localized variations in material properties and redepositing of ablated material. However, the combination of high-pulse scribes and precision-controlled lower-power scribes help mitigate these effects, ensuring reliable electrical isolation. The observed edge blurring may result from localized heat diffusion, leading to a gradual transition rather than a sharp boundary. However, this does not significantly impact electrical performance, as confirmed by our characterization results. The final scribing steps help refine the edges, minimizing series resistance effects.

While the P2 region morphology can influence contact resistance, our optimization strategy ensures that any potential resistance increase remains within an acceptable range. Our electrical characterization (e.g., IV curves, electroluminescence imaging) confirms effective isolation and stable module performance.

To further support this explanation, we have attached an image (Fig. S3) illustrating the laser scribing strategy and its impact on the ablation profile (panel a)). Additionally, we provide further photos with higher resolution, specifically showing a close-up view of the P1 and P2 regions for your consideration(panel b)).

Immagine che contiene testo, bianco e nero, calcolatore, arte

Il contenuto generato dall'IA potrebbe non essere corretto.

Figure S3: a) Part of the P2 ablation matrix used to optimize ablation parameters., b) Close-up view of the P1 and P2 ablation areas. Both images are constrained by the camera’s field of view

Table S1reports the PV characteristics of the best devices shown in Fig. 2e in the main text.

Table S1 electrical parameters of the best devices according to their active area

Fig. S4 shows the statistical trend of the FF as a function of the active area, calculated on 5 samples, where the error bars representing one standard deviation. It can be observed that for devices with active areas of 1 cm² and 10 cm², as well as for those with 68 cm² and 150.4 cm², the FF remains comparable within the margin of error. The overall decrease in FF with increasing active area is attributed to the rise in the number of interconnected cells, from a single cell (without series interconnections) in the 1 cm² case to 5, 17, and 24 cells in the 10 cm², 68 cm², and 150.4 cm² cases, respectively. This increase in interconnections leads to higher overall series resistance. Additionally, the larger cell dimensions may exacerbate the impact of ablation-related defects during the P2 process, further contributing to FF reduction.

Figure S4: Statistical trend of the module FF as a function of the active area, evaluated over five samples for each active area dimension.

PSMs fabrication 

The same stack and process were used for module fabrication. High-temperature-resistant silver paste was printed on the busbar before the spray pyrolysis process to avoid blocking charge extraction by the compact TiO2 layer. A 0.7 cm free space was left on the substrate edge for the lamination process. Further details about different PSMs are reported in Table S2.

Table S2 Material Quantities and Process Parameters for Upscaling Perovskite Solar Cells

| Device (cm2) | Active area (cm2) | Meso.TiO2 (mL) & speed (rpm/s) | Perovskite (mL) & Speed (rpm/s) | CB (mL) | PEAI (mL) & Speed (rpm/s) | PTAA (mlL) & Speed (rpm/s) |
| --- | --- | --- | --- | --- | --- | --- |
| 6.25 | 1 | 0.12 [3000] | 0.09 [5000] | 0.1 | 0.1 [2500] | 0.09 [3000] |
| 31.36 | 10 | 0.5 [3000] | 0.45 [5000] | 0.5 | 0.5 [2500] | 0.45 [3000] |
| 121 | 68 | 0.95 [3000] | 0.7 [4000] | 0.8 | 0.8 [2500] | 0.8 [3000] |
| 243.36 | 150.4 | 1.4 [3000] | 1.2 [3500] | 1.5 | 1.5 [2500] | 1.4 [2300] |

Thermal stability

The thermal stability of the encapsulated PSMs was tested according to the ISOS-D2 protocol (dry heat at 85°C). The starting point of the test (t = 0), when we recorded the initial PCE, was immediately after the encapsulation process. The PCE loss observed after the encapsulation of PTAA-based PSMs was attributed to both thermal stress during the lamination process [1] and mechanical stress originated by the mismatch of thermal expansion coefficients of the materials composing the cell stack [2]. As shown in Figure S5, the PTAA-based PSM reached a T80 after 1250 h. After 1100 h, the device retained approximately 84% of its initial PCE.

|  |  |
| --- | --- |

Figure S5. a) ISOS-D2 stability test (PCE vs. time) for the investigated PSMs. b) Visual inspection of the PSMs after 3-month ISOS-D2 stability test.

Workflow of Perovskite solar panel manufacturing 

Figure S6 outlines the entire Perovskite solar panel (PSP) manufacturing chain in 17 steps, subdivided into 14 steps for fabricating the PSMs, followed by 7 processes for their integration into the PSPs [3].

Immagine che contiene testo, schermata, Carattere, Rettangolo

Il contenuto generato dall'IA potrebbe non essere corretto.

Figure S6: Workflow of PSP manufacturing chain [3].

Perovskite solar panel architecture

In the realm of perovskite solar technology, the configuration of solar panels plays a pivotal role in determining their overall performance, durability, and practicality.[3] A modular approach, wherein the panel comprises interconnected smaller perovskite modules, offers several advantages over a single large perovskite solar module. Firstly, the modular approach allows to enhance reliability and fault tolerance, in fact, any damage, degradation, or shading affects only the specific module impacted, leaving the rest of the panel operational. Conversely, in a single large module, a defect could compromise the entire panel's functionality. This inherent fault tolerance in modular designs ensures sustained energy production even when individual modules encounter issues. Moreover, smaller modules facilitate better heat dissipation, which is crucial for maintaining the stability and efficiency of perovskite materials. Efficient thermal management reduces the risk of thermal degradation, thereby enhancing the long-term stability of the solar panel. Regarding scalability and manufacturing, the modular approach allowing for customized panel sizes tailored to specific energy requirements. Additionally, smaller modules are easier to manufacture and quality-test before assembly, minimizing production defects. This approach aligns with existing manufacturing processes, making the integration of perovskite modules more feasible on an industrial scale.[4]

Manufacturers can produce standardized modules that can be combined in various configurations to meet specific energy requirements or spatial constraints. This flexibility is particularly beneficial in residential or commercial installations where space and energy needs vary. For the sake of clarity, under ideal conditions—without shading, with uniform illumination, constant temperature, and no degradation—a single large module might achieve slightly higher efficiency than a modular panel. This is because it eliminates efficiency losses associated with electrical interconnections between smaller modules, such as contact resistance and bypass diodes. In such conditions, a single large module could marginally outperform a modular setup by maximizing the active surface area and minimizing interconnection losses. Furthermore, the absence of interconnection-related losses in a single-module design could lead to a performance gain of approximately 1–2%, attributed to improved interconnection quality and material integrity. However, the layout of the cells constituting the module could represent a limiting factor for the final module efficiency. Indeed, enlarging the module dimension forces an increasing number of the constituting cells to limit the single cell active area, thereby limiting the single cell current and eventually the associated resistive loss over the vertically interconnections. This induces a increase of the overall module series resistance with a consequently FF penalization.

Considering these factors, modular perovskite solar panels present a more practical and resilient solution for real-world applications compared to single large modules.

Outdoor measurement

The fluctuation in VOC observed in Figure 6d is due to variations in environmental conditions during the outdoor measurement. Factors such as temperature fluctuations, changes in solar irradiance, and minor shading effects can contribute to small variations in VOC over time. Additionally, the stabilization process of the PSP under continuous operation can also cause a gradual increase in Voc. Despite these minor variations, the VOC stabilizes around 87 V, demonstrating the reliable performance of the device under MPP tracking.

In particular, the light soaking experiment was performed to investigate the dependence of VOC, on light illumination, until the electrical characteristics was stabilized. The measurement was conducted during midday at Crete in August to maintain a nearly stable solar irradiance of 1000±30 W∙m-2 and the fluctuations of the VOC in Fiure. 6d (reported in the main text) are partly due to the fluctuations of the irradiance and the temperature because the panel was still exposed to the surrounding environmental conditions. Figure S7 shows also the solar irradiance and the panel temperature at the time of the measurement.

Figure S7: The solar irradiance and the panel temperature in the time of the measurement (midday in Crete in August)

Electrochemical impedance spectroscopy analysis of the PSP. 

Figure S8 reports the electrochemical impedance spectroscopy (EIS) analysis of the PSP, showing Nyquist plots for a representative PSP as a function of negative and positive voltage bias (Vbias) . When applied voltages (Vbias) are more negative than -0.4 V, the panel's bypass diodes activate. As the voltage increases, the charge transfer resistance (Rct) decreases, following the same trend for positive voltage bias, as indicated by the arc diameter.

Immagine che contiene testo, schermata

Il contenuto generato dall'IA potrebbe non essere corretto. 

Figure S8. EIS analysis of the PSP for a) negative and, b) positive voltage bias

For further details in EIS for solar panels, G(s) typically represents the system's transfer function in the frequency domain. It describes the relationship between the input perturbation (often a small AC voltage) and the output response (the resulting AC current), providing insights into the dynamic behaviour of the system. As depicted in Figure S9, the G(S) increases with increasing frequency. With increasing voltage, G(S) decreases until it reaches -1 volt, after which it remains constant at zero.

Immagine che contiene testo, schermata, Carattere

Il contenuto generato dall'IA potrebbe non essere corretto.

Figure S9. The EIS analysis of the PSP for the transfer function of system G(S), as a fucntion of voltage and frequency.

The infrared camera was used to capture the thermal image and heat distribution within the PSM. This test highlights how sunlight affects various parts of the PSP during field testing, showing a temperature increase up to 85°C during sun exposure, as depicted in Figure S10.

Immagine che contiene testo, schermata, quadrato

Il contenuto generato dall'IA potrebbe non essere corretto.

Figure S10. a) PSP position in field test. b) Heat distribution within PSP, as measured with infrared camera. c) Different heat tension across the PSP. d) Heat distribution of junction box backside of the panel.

References

- Rombach, F.M., S.A. Haque, and T.J. Macdonald, Lessons learned from spiro-OMeTAD and PTAA in perovskite solar cells. Energy & Environmental Science, 2021. 14(10): p. 5161-5190.

- Tang, J., et al., Non-destructive Single‐Glass Vacuum Lamination Encapsulation for Perovskite Solar Cells with Long‐Term Stability. Solar RRL, 2024. 8(2): p. 2300801.

- Pescetelli, S., et al., Integration of two-dimensional materials-based perovskite solar panels into a stand-alone solar farm. Nature Energy, 2022. 7(7): p. 597-607

- Pengchen Z., et al., Toward the Commercialization of Perovskite Solar Modules, Advanced Materials, 2024 36 (15): p. 2307357
